# Enrichment of superoxide dismutase 2 in glioblastoma confers to acquisition of temozolomide resistance that is associated with tumor-initiating cell subsets

**DOI:** 10.1186/s12929-019-0565-2

**Published:** 2019-10-19

**Authors:** Chia-Hung Chien, Jian-Ying Chuang, Shun-Tai Yang, Wen-Bin Yang, Pin-Yuan Chen, Tsung-I Hsu, Chih-Yuan Huang, Wei-Lun Lo, Ka-Yen Yang, Ming-Sheng Liu, Jui-Mei Chu, Pei-Hsuan Chung, Jr-Jiun Liu, Shao-Wen Chou, Shang-Hung Chen, Kwang-Yu Chang

**Affiliations:** 1 National Institute of Cancer Research, National Health Research Institutes, 367 Sheng-Li Road, Tainan, 70456 Taiwan; 2 Center for Neurotrauma and Neuroregeneration, Taipei Medical University, Taipei, Taiwan; 3 The Ph.D. Program for Neural Regenerative Medicine, Taipei Medical University, Taipei, Taiwan; 4 Division of Neurosurgery, Shuang-Ho Hospital, Taipei Medical University, Taipei, Taiwan; 5 Graduate Institute of Medical Sciences, College of Medicine, Taipei Medical University, Taipei, Taiwan; 6 Department of Neurosurgery, Chang Gung Memorial Hospital at Keelung, Keelung City, Taiwan; 7 School of Medicine, Chang Gung University, Taoyuan, Taiwan; 8 Department of Neurosurgery, Chang Gung Memorial Hospital at Linkou, Taoyuan City, Taiwan; 9 Division of Neurosurgery, Department of Surgery, National Cheng Kung University Hospital, Tainan, Taiwan; 10 Department of Neurosurgery, Kaohsiung Chang Gung Memorial Hospital and Chang Gung University College of Medicine, Kaohsiung, Taiwan; 11 Division of Hematology/Oncology, Department of Internal Medicine, National Cheng Kung University Hospital, College of Medicine, National Cheng Kung University, Tainan, Taiwan

**Keywords:** SOD2, ROS, Glioblastoma, Tumor-initiating cells, Temozolomide

## Abstract

**Background:**

Intratumor subsets with tumor-initiating features in glioblastoma are likely to survive treatment. Our goal is to identify the key factor in the process by which cells develop temozolomide (TMZ) resistance.

**Methods:**

Resistant cell lines derived from U87MG and A172 were established through long-term co-incubation of TMZ. Primary tumors obtained from patients were maintained as patient-derived xenograft for studies of tumor-initating cell (TIC) features. The cell manifestations were assessed in the gene modulated cells for relevance to drug resistance.

**Results:**

Among the mitochondria-related genes in the gene expression databases, superoxide dismutase 2 (SOD2) was a significant factor in resistance and patient survival. SOD2 in the resistant cells functionally determined the cell fate by limiting TMZ-stimulated superoxide reaction and cleavage of caspase-3. Genetic inhibition of the protein led to retrieval of drug effect in mouse study. SOD2 was also associated with the TIC features, which enriched in the resistant cells. The CD133^+^ specific subsets in the resistant cells exhibited superior superoxide regulation and the SOD2-related caspase-3 reaction. Experiments applying SOD2 modulation showed a positive correlation between the TIC features and the protein expression. Finally, co-treatment with TMZ and the SOD inhibitor sodium diethyldithiocarbamate trihydrate in xenograft mouse models with the TMZ-resistant primary tumor resulted in lower tumor proliferation, longer survival, and less CD133, Bmi-1, and SOD2 expression.

**Conclusion:**

SOD2 plays crucial roles in the tumor-initiating features that are related to TMZ resistance. Inhibition of the protein is a potential therapeutic strategy that can be used to enhance the effects of chemotherapy.

**Graphical abstract:**

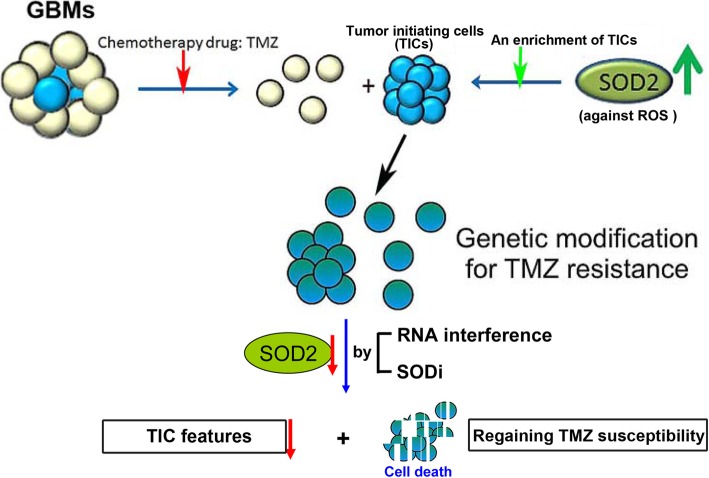

## Background

Glioblastoma (GBM) is a fatal disease with a mean survival of approximately only one year, even with comprehensive treatment [1]. Currently, unlike other cancers, only limited therapeutic agents are available for the control of this disease. The difficulty in therapeutic development is often due to the blood brain barrier as a natural obstacle to drug penetration and the tendency for the tumor to develop resistance. For example, GBM acquires resistance to temozolomide (TMZ), which is one of the best-recognized chemotherapeutic agents to be introduced against GBM [2]. This drug acts through the induction of lethal DNA damage and the subsequent production of radical oxygen species (ROS) [3], but the resulting control is mostly only short-term, as up to 90% of patients who undergo surgical resection are expected to experience disease recurrence [4]. The remaining disease course is often poor, as the tumor then exhibits a much more dismal nature compared with the original one. The factors that lead to the ominous features are unclear. Thus far, pre-existing O^6^-methylguanine-DNA methyltransferase (MGMT) is the only known single contributory gene that has been clearly described [5]. This gene is associated more with innate resistance, and together with other DNA repair genes, might serve as a predictor of drug response [6]. Much uncertainty exists in regard to the ability of cells to acquire resistance due to the complicated and multifactorial mechanisms involved in TMZ resistance.

An emerging concept in cancer biology suggests that specific subpopulation of cells has a greater survival advantage in a challenging environment and that they maintain their ability to form a tumor and become resistant to therapy [7]. These cells, referred to as tumor-initiating cells (TICs) or cancer stem-like cells, are characterized by exhibiting self-renewal, multipotency, and other TIC features associated with normal stem cell properties [8]. The cells tend to withstand standard therapies and are associated with poor treatment outcomes. In GBM, the presence of TIC provides an additional explanation of the capability of tumors to withstand and survive TMZ toxicity regardless of MGMT status. Supportively, cells carrying the stemness gene Nestin could initiate the recurrence of GBM following TMZ treatment [9]. We previously reported the acquisition of TMZ resistance after long-term treatment with the drug enriches the TIC features [10]. Interestingly, a recent study of clinical specimens has also suggested the presence of glioma stem cells as a single factor associated with a poor prognosis [11].

Altered metabolic reprogramming by mitochondrial control in cancer may play a role in chemoresistance [12]. For example, strict regulation of ROS levels is essential for the maintenance of cell viability and to avoid oxidative damage from stress overload [13]. In fact, excessive ROS is harmful to cells and excessive ROS production is known in part contributing to the cytotoxic effects of TMZ [3]. In our previous study, we identified dysregulation of Sp1 contributes to the tolerance of TMZ-induced ROS in TMZ-resistance cells [14]. The transcriptional factor Sp1 was found to modulate superoxide dismutase 2 (SOD2 or MnSOD) expression, which is known to function in mitochondria to regulate oxidative stress and energy metabolism [15]. This protein, other members of the SOD, catalase, and glutathione peroxidase family, are ROS scavengers. The expression of SOD2 is crucial for the development of cerebral cortex as it regulates the levels of ROS, which determines the fate of neuronal progenitor cells [16]. Its expression is also generally increased in brain cancer as compared with normal brain [14]. Despite a basic understanding of this protein, less is known about its impact on the disease course of GBM.

Given that the tumor cells with TIC features are prone to withstand treatment, and given the crucial role of SOD2 in the regulation of ROS, we hypothesized that up-regulation of SOD2 is important for GBM to acquire TMZ resistance and is associated with enhancement of the TIC features.

## Methods

### Gene expression analysis

Gene expression profiling was performed on RNA samples from parental and resistant cell lines using microarray (U87MG) or RNA-seq (A172). The list of 1174 mitochondria-related genes was constructed according to MitoCarta2.0 [17] and Mitochondria RT2 Profiler PCR Array (Qiagen, Denmark). The mitochondria-related genes that were significantly different between parental and TMZ-resistant cells were identified by at least a 1.5-fold difference and a *P*-value< 0.05. The heat map was generated using Multi-Experiment Viewer (http://mev.tm4.org/) according to the log2 (fold change) values of selected genes.

### Analysis of the Cancer genome atlas (TCGA) clinical datasets

For gene expression analysis, the GBM clinical transcriptome next-generation sequencing (NGS) data were obtained from TCGA database (https://portal.gdc.cancer.gov/). This included a total of 169 specimens, which consisted of 156 primary and 13 recurrent tumors. The data of fragments per kilobase of transcript per million mapped reads upper quantile (FPKM-UQ) were downloaded for further processing. The expression fold change and significance level (t-test) of mitochondria-related gene expression between primary and recurrent GBM NGS data were calculated. For survival, a publicly available cancer microarray database, SurvExpress, was used for analysis of the TCGA datasets [18].

### Culture of the GBM cell lines and derivation of the TMZ-resistant cells

The human GBM cell lines U87MG and A172 were purchased from American Type Culture Collection (Manassas, VA, USA). DMEM (Thermo Fisher Scientific, Waltham, MA, USA) with supplementation of 10% serum and antibiotics was used as medium. The resistant cells were derived from long-term co-incubation with 100 μM TMZ (Sigma-Aldrich, St. Louis, MO, USA) which significantly reduced cellular proliferation and survival in the beginning, but recovered eventually. Random single-cell clones were then cultured, with majority having SOD2 expression [14]. Analysis of the clones showed an association between SOD2 and the TIC biomarkers (Additional file 1: Figure S1A). We selected r#10 of U87MG (U87MG-r#10) and r#6 of A172 (A172-r#6) for the following studies. Co-incubation with TMZ was kept for regular maintenance of the resistant cells.

### Construction of patient-derived xenograft models for primary tumor study

Samples were obtained directly from the tumor tissue, which were surgically resected in a naïve GBM patient (GBM#4) and a recurrent GBM patient with prior multiple treatment (GBM#1). They were then minced and implanted into subcutaneous area of immunocompromised NOD-SCID mice (BioLASCO, Taipei, Taiwan) in less than 6 h. After tumor formation, the mice were sacrificed for extraction of the tumor, annotated as P0. The tumor was minced and successively implanted into another mouse for the first passage, annotated as P1. The passage continued for maintenance of the primary cells. For cell study or for cryopreservation, tumors of only three or fewer passages were applied to keep the tumor features [19]. The tumor samples were minced and incubated in a papain dissociation kit (#130–095-942, Miltenyi Biotec, Bergisch Gladbach, Germany) for treatment with gentleMACS™ dissociators. The GBM cells were then isolated by negative magnetic selection using the Mouse Ablation Kit (#130–104-694, Miltenyi Biotec). The processed cells can be used for sphere cell cultures (in serum-free medium: DMEM/F12 (Thermo Fisher Scientific), 1% penicillin/streptomycin, 2% B27 (Thermo Fisher Scientific), 10 ng/ml basic fibroblast growth factor (Cell Guidance Systems, Cambridge, United Kingdom), and 10 ng/ml epidermal growth factor (ProSpec, East Brunswick, NJ, USA)), cell sorting and animal experiments. We also cultured the cells in low-serum (1–2%) containing medium for short-term duration to observe cell morphology and growth [20, 21].

### Tumor spheroid formation assays

Spheroid cells were cultured with serum-free medium containing 0.3% methylcellulose (Sigma-Aldrich) in ultra-low adherent plates [22]. For the tumor spheroid formation assay, low numbers of cells (e.g., 1, 5, 10, 20, and 50 cells) were applied. After 2 weeks, the formation of spheres and their diameters was assessed. The frequency of initiation capacity was then calculated using Extreme Limiting Dilution Analysis (ELDA, http://bioinf.wehi.edu.au/software/elda/).

### Western blot analysis

The cell lysate was separated via SDS-PAGE and transferred onto polyvinylidene difluoride membranes (Bio-Rad, Hercules, CA, USA). The membranes were first blocked with 5% nonfat milk and were then incubated overnight with primary antibodies against SOD2 (1:3000, Cell Signaling, Danvers, MA, USA), CD133 (1:1000, Proteintech, Rosemont, IL, USA), Bmi-1 (1:1000, GeneTex, Irvine, CA, USA), SOX2 (1:1000, GeneTex), Oct4 (1:5000, GeneTex), caspase 3 (1:1000, Cell Signaling), Oct3/4 (1:1000, Santa Cruz, Dallas, TX, USA), vimentin (1:1000, GeneTex), MGMT (1:1000, BD, Franklin Lakes, NJ, USA), and beta-actin (1:5000, Millipore, Burlington, MA, USA). After the membranes were washed, they were incubated with secondary antibodies. Finally, after eliciting signals with chemiluminescence substrate, Amersham Hyperfilm ECL (GE Healthcare, Chicago, IL, USA) was used to detect the expression intensity. The density was quantified by ImageQuant (GE Healthcare).

### Immunohistochemistry (IHC)

All paraformaldehyde-fixed, paraffin-embedded tissue sections were prepared from xenograft mouse or human archival tissue (Pathology Department of National Cheng Kung University Hospital). The method of staining was previously described [14]. The primary antibodies were described in the western blot analysis but at a dilution of 1:200. The staining were automatically identified and assessed by ImageJ (http://rsbweb.nih.gov/ij/).

### Quantitative real-time polymerase chain reaction (qRT-PCR)

Total RNA was isolated by TRIzol (Invitrogen, Carlsbad, CA, USA) following a standard procedure and was subjected to qRT-PCR with SuperScript II reagent (Invitrogen). The product was mixed with SYBR® Green Master Mix (Applied Biosystems, Foster City, CA, USA); the specific primers used were as follows: (SOD2, F:5′-GGCCTACGTGAACAACCTGAA, R:5′-CTGTAACATCTCCCTTGGCCA; CD133, F:5′-TCCACAGAAATTTACCTACATTGG, R:5′-CAGCAGAGAGCAGATG.

ACCA; Bmi-1, F:5′-TGGAGAAGGAATGGTCCACTTC, R:5′-GTGAGGAAACTGT.

GGATGAGGA; SOX2, F:5′-AAATGGGAGGGGTGCAAAAGAGGAG,R:5′-CAGCT.

GTCATTTGCTGTGGGTGATG; GAPDH, F:5′-GAAGGTGAAGGTCGGAGTC, R:5′-GAAGATGGTGATGGGATTC). The expression was detected using an ABI 7000 Sequence Detection System (Applied Biosystems) and was normalized to GAPDH using the 2^-ΔΔCT^ formula.

### Clonogenic assay and cell density assay

For clonogenic assay, 400 cells/well were cultured in 6-well plate with treatment given on the next day. The cells were washed after three days and incubated in treatment-free medium to allow them to grow into colonies. They were then stained and fixed with 50% ethanol containing 0.5% methylene blue for 90 min, and the numbers of the colonies were counted. For cell density assay, 5000~20,000 cells/well were cultured in 6-well plate with treatment given on the next day. They were allowed for proliferation for three days. The cells were then stained and fixed with the aforementioned solution, and was dissolved in 1% N-lauroyl-sarcosine followed by measurement of the optical density at 570 nm.

### Detection of surface CD133 in cells and mitochondrial ROS expression by flow cytometry

Cells were dissociated and labeled with APC-anti-CD133 (Miltenyi Biotec) to detect the stemness feature or MitoSOX® (Invitrogen) to detect mitochondria-specific ROS expression. The staining procedure followed the manufacturers’ protocols with minimal adjustments. Fluorescence-activated cell sorting (FACS) was applied using a FACSCalibur system (BD) and CellQuest software (BD) for data collection and analysis, including determination of mean fluorescent intensity (MFI). For sorting, a FACSAria™ III (BD) was used to isolate the CD133 specific cells. The sorted CD133^+^ and CD133^−^ cells were collected and cultured in the serum-free medium and the serum-containing medium, respectively.

### Measurement of SOD2 activity

The activity of SOD2 was detected using an Amplex Red Hydrogen Peroxidase Assay (Invitrogen) and a Superoxide Dismutase Assay Kit (Cayman Chemical, Ann Arbor, MI, USA) according to the manufacturers’ instructions. Briefly, the cells were cultured in plates with or without treatment and were then transferred to a microplate. The indicated volume of working solution was pipetted into each well to initiate the reaction. To detect SOD2 enzyme activity, potassium cyanide was added simultaneously to block SOD1 and SOD3 reactions [23]. A microplate reader with the indicated excitation/emission wavelengths was used for data collection and analysis.

### RNA-based gene modulation of SOD2

Lipofectamine® RNAiMAX reagent (Invitrogen) and LTX with Plus™ reagent (Invitrogen) were used following manufacturer’s protocol for transient transfection of SOD2 siRNA (S13268, Ambion, Austin, TX, USA) and pBI-EGFP-MnSOD (#16612, Addgene, Cambridge, MA, USA), pBI-EGFP (kindly provided by Dr. Hsiao-Sheng Liu, National Cheng Kung University, Taiwan), respectively. For stable knockdown, the cells were infected with SOD2-lentiviral short hairpin RNA (shRNA) or empty vector (both from RNAi Core, Academia Sinica, Taiwan). The next day, the infected cells were then selected for stable clones in antibiotic-containing medium for weeks, which was followed by confirmation of the knock-down efficiency and selection (Additional file 1: Figure S1B).

### Xenograft mouse model for tumor growth assessment or survival studies

Male NOD-SCID mice 5~6 weeks of age were used in this study. For the tumor growth assessment, cells (2 × 10^6^) were inoculated into the subcutaneous area of the right flank. The tumor volume was measured twice a week according to the following National Cancer Institute formula: length×width^2^ × 3.14/6. When the tumors reached 200 mm^3^ in size, the animals were randomly assigned for treatment. For the survival studies, burr holes were generated in the right frontal brain area of the skull. Then, using a stereotactic instrument, the cells (2.5 × 10^5^) were injected through an ultrafine needle at a location 1.5 mm anterior to the bregma, 2.5 mm lateral to the midline, and 3.5 mm ventral to the surface of the dura mater. Treatment was initiated after 5 days. Drug administration consisted of TMZ (5 mg/kg) via oral gavage and/or the SOD inhibitor sodium diethyldithiocarbamate trihydrate (DETC, 100 mg/kg, Sigma-Aldrich) via intraperitoneal injection. TMZ was administered 3–4 h after the DETC injection.

### In vivo assessment of the TIC tumorigenic potential

A titrated number of tumor cells was injected subcutaneously into the NOD-SCID mice. The tumor volume was measured regularly, and the frequency of stem cell initiation was analyzed using ELDA. The tumor was then extracted and subsequently isolated for serial transplantation into another mouse. Gene expression during each passage was assessed by qPCR after tumor extraction.

### Statistics

Data were statistically analyzed using Prism 7 (GraphPad, La Jolla, CA, USA). The differences in continuous variables were calculated by unpaired, two-tailed Student’s t-test. The survival data were plotted by Kaplan-Meier curve, and the difference was calculated using the Log-Rank test. Significance was set at *P* ≤ 0.05.

## Results

### SOD2 expression was associated with TMZ resistance in GBM

**Fig. 1:**
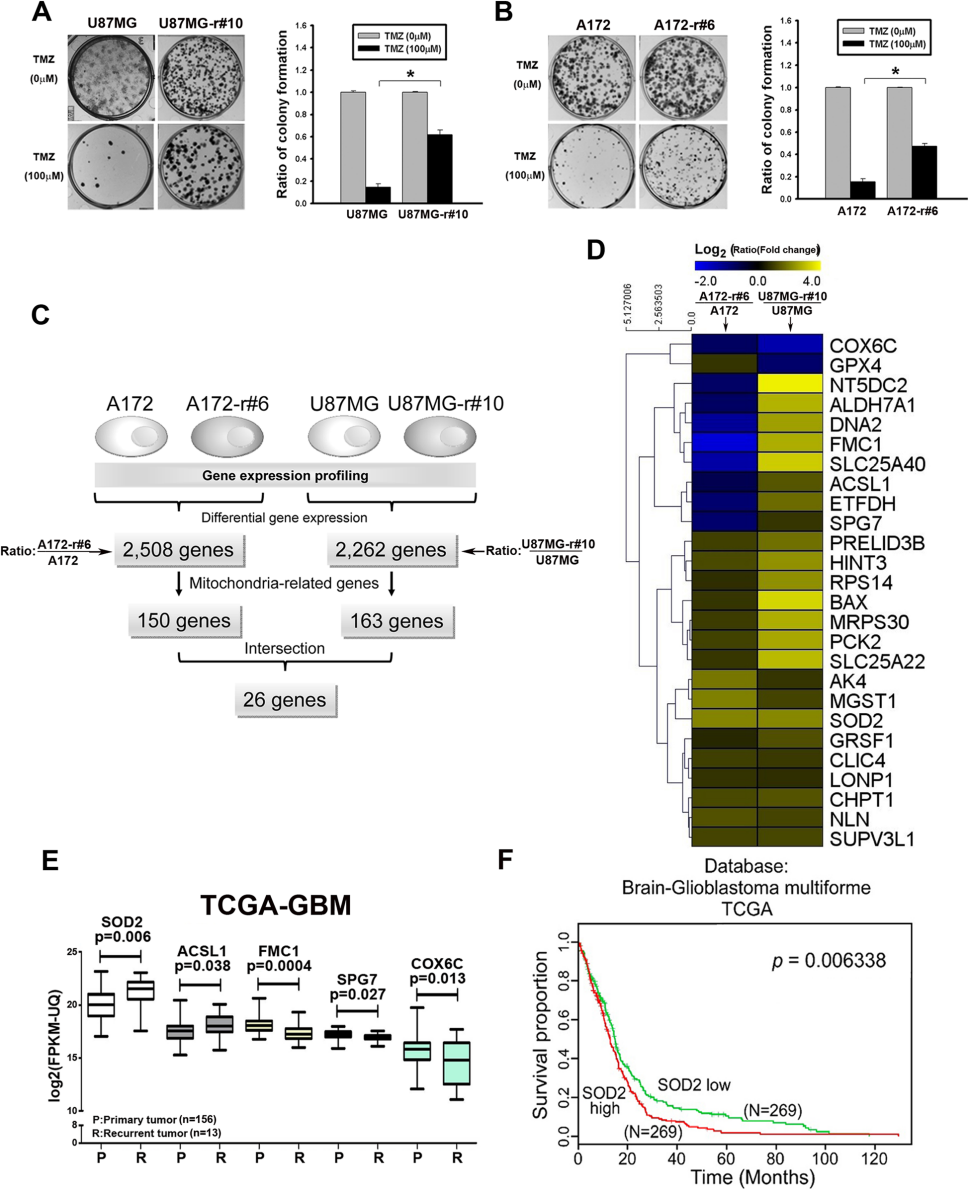
Analysis of TMZ-resistant cell lines and identification of resistance-associated genes. **a**&**b** Clonogenic assay of the parental and resistant U87MG cells (**a**, r#10 as the resistant clone) and A172 (**b**, r#6 as the resistant clone). Cells were treated for 3 days and cultured until Day 7 (*P < 0.05). **c** Flowchart of differentiating the mitochondria-related genes that significantly differed in TMZ-resistant cells. In U87MG- and A172-resistant cells, 26 overlapping mitochondria-related genes were identified. **d** Heatmap shows the significance of the 26 genes in the two resistant cell lines. **e** Validation of these 26 candidative mitochondrial genes in the TCGA-GBM dataset was analyzed. Only the 5 genes which expression was statistically significant were shown. **f** Kaplan-Meier curves of the TCGA databases from SurvExpress [18]. Each line refers to cases in which SOD2 gene expression was higher or lower than the median

To explore the critical factors in treatment resistance, cell models of acquired resistance were derived from U87MG and A172 cells (U87MG-r#10 and A172-r#6, respectively) [14]. A significantly higher number of colonies were noted in the resistant cells when they were co-cultured with TMZ, which reveals their ability to survive the drug toxicity (Fig. 1a and b). An array-based analysis of gene expression differentiated 2508 and 2262 genes between the parental and the resistant A172 and U87MG cells, respectively (Fig. 1c). Among them, 150 and 163 genes are mitochondria-related genes, which later showed an overlap of 26 significant genes in these two cohorts (Fig. 1c and d). We checked the clinical significance of these 26 genes using the TCGA dataset, with only five of them statistically differed in the recurrent versus naïve tumor (Fig. 1e). SOD2 was the most significant gene that had inferior survival curves with high expression in TCGA, which was in agreement with worse biological implication from the cell line studies (Fig. 1f for TCGA and Additional file 2: Figure S2 for the other datasets to support).

### SOD2 promotes TMZ resistance in GBM cells

**Fig. 2:**
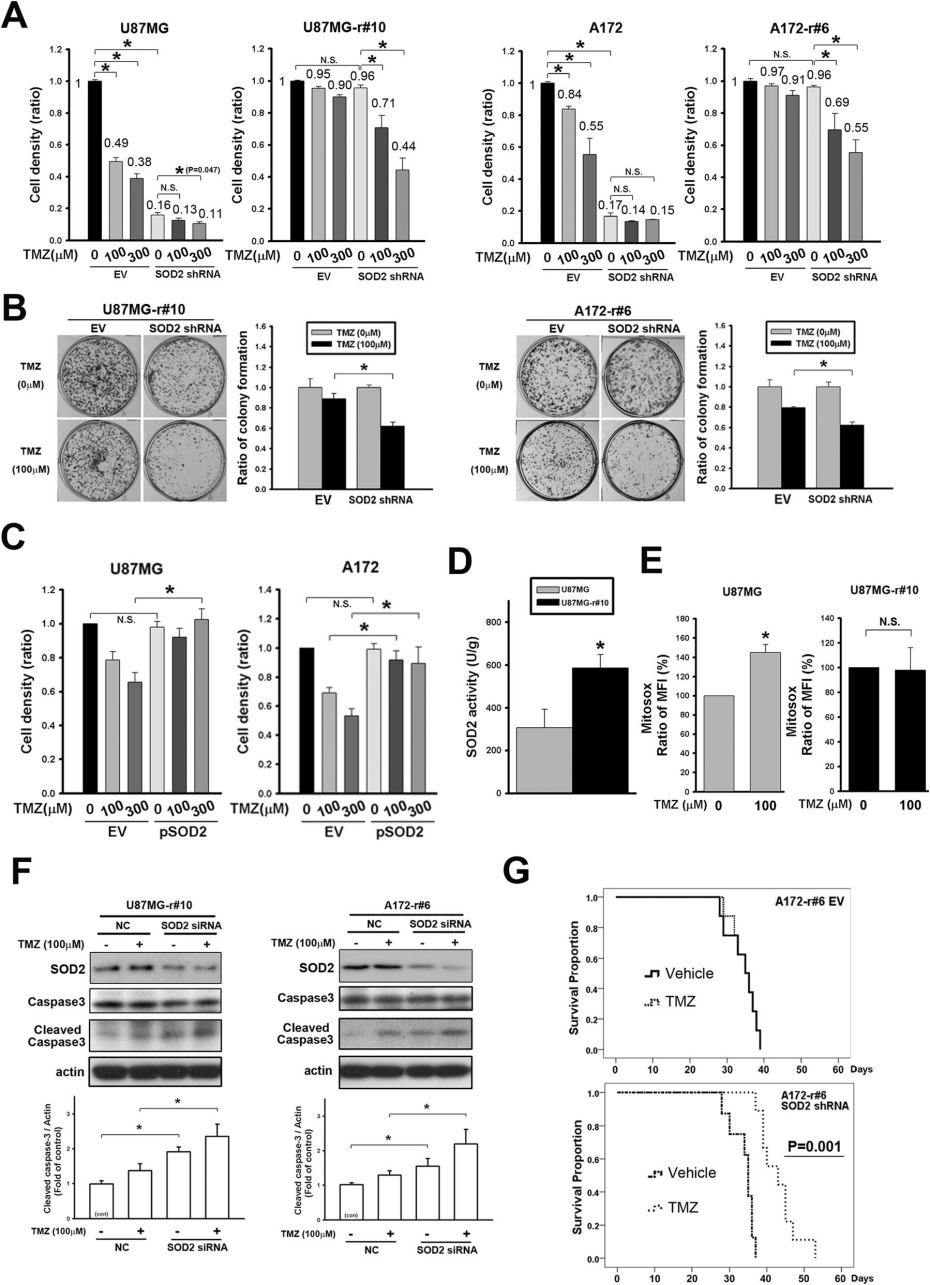
The resistant features in cell lines were related to increased SOD2 expression. **a** Cell density assay of the parental and resistant U87MG and A172 cells with SOD2 knockdown or empty vector (EV) control groups. The cells were treated with TMZ in dose-dependent manner for 72 h. **b** Clonogenic assay of the resistant cell lines with stable SOD2 knockdown. TMZ was given after cell attached, incubated for 72 h and then changed to drug-free medium for colony to form. The bar graphs showed the ratio of TMZ-treated groups to the untreated control of their own cells. **c** Cell density assay of the parental cell lines with overexpression of SOD2. The cells were treated with TMZ in dose-dependent manner for 72 h. **d** The basal level of SOD2 activity was examined in the parental and the resistant U87MG. The bar graph represented mean values of triplicate experiment. **e** The TMZ-induced mitochondrial ROS were detected with MitoSox in parental and resistant U87MG cells. The mean fluorescent intensity (MFI) gated by the control was calculated into the ratio over the parental cells. The average of triplicate experiment was shown in the bar graphs. **f** The caspase 3 expression was detected in the resistant cell lines after TMZ incubation for 24 h. Transfection with SOD2 siRNA was done 72 h before TMZ treatment. **g** Survival curves of the orthotopic mouse model in which mice were implanted with lentiviral empty vector (EV)- or SOD2 shRNA-infected resistant A172 cells (*n* = 8 for each group). Treatment with TMZ or vehicle was administered daily, five days a week (*P* = 0.001 in the SOD2 shRNA groups, *P* > 0.05 in the EV groups). (*P < 0.05; N.S., non-significant)

To confirm the crucial role of SOD2 in cell resistance, RNA interference (RNAi) was applied to knock down the gene expression. We first investigated its impact by cell density assay (Fig. 2a). In those carrying the shRNA, the result showed lower ratio of cell density in resistant cells comparing to the parental ones with TMZ treatment (0.74- versus 0.80-fold and 0.46- versus 0.67-fold, respectively, for 100 μM and 300 μM in U87MG; 0.72- versus 0.81-fold and 0.57- versus 0.88-fold, respectively, for 100 μM and 300 μM in A172). On the other hand, only less than 10% loss for cell density was observed in control groups of the resistant cells when TMZ was given, while much larger loss was noted for those of the parental cells. The long-term effect of survival was also studied because, unlike the limited short-term impact with cell density assay, TMZ still affected the colonies formation of the resistant cells but in a less extent comparing to the parental cells (Fig. 1a) [24]. Hence, the clonogenic assay showed that drug toxicity remained noted in the antibiotic-selected control resistant cells, and was even enhanced in the SOD2 knockdown groups (Fig. 2b). On the contrary, overexpression of SOD2 in the parental cell lines resulted in higher cell density with TMZ, suggesting resistance against the drug (*P* < 0.05 for 100 μM in A172 and for 300 μM in U87MG and A172, Fig. 2c).

In regard with the altered antioxidant capacity of the cells to adapt intrinsic oxidative stress that led to resistance [25], SOD2 function was assessed. The increased activated SOD2 function (Fig. 2d) was associated with less stimulated mitochondrial ROS level 24 h after co-incubation with TMZ in resistant cells (Fig. 2E & Additional file 3: Figure S3A, the treatment changes MFI to the ratio of 145.1 and 98.1% comparing to their own untreated groups in parental cells and in resistant cells, *P* = 0.005 and 0.92, respectively). The impact to the cells was studied by cleaved caspase 3 expression, which was significantly induced when the knockdown cells were coincubated with TMZ compared with the control in resistant cell lines (Fig. 2f, and supportively, with adherent resistant primary tumor cells GBM#1 in Additional file 4: Figure S4). In addition, stable downregulation of SOD2 in the resistant cells but not in the parental cells led to the longer survival time of orthotopic xenograft mice that were treated with TMZ, supporting its pivotal role in cells that acquire TMZ resistance (Fig. 2g).

### Enriched SOD2 was featured in enhanced TICs

**Fig. 3:**
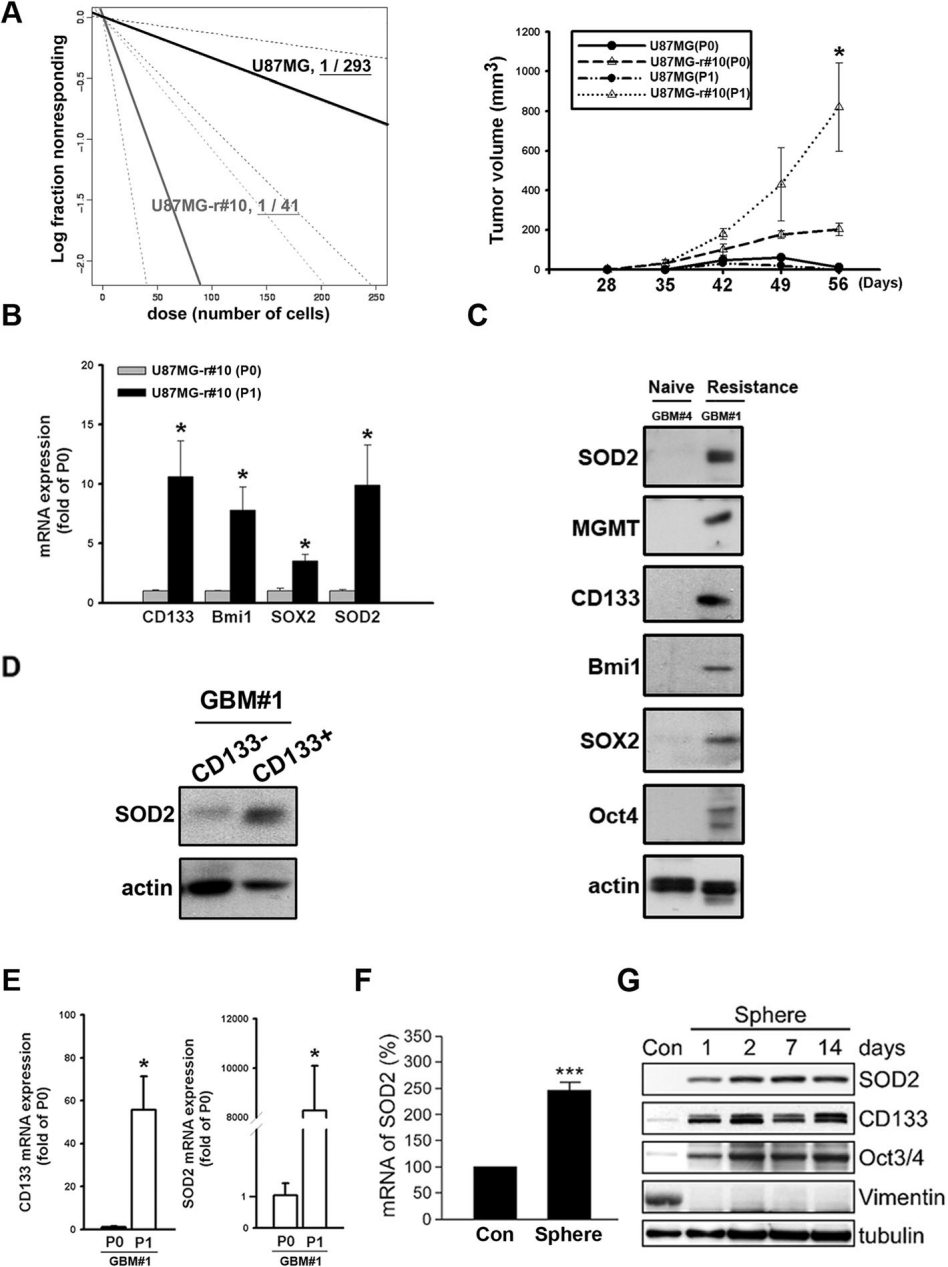
Enrichment of the tumor-initiating cell (TIC) features in TMZ-resistant cells enhanced SOD2. **a** The estimated stem cell incidence by in vivo extreme limiting dilution assay after subcutaneous injection of parental or resistant cells (left). The tumor size measurement of the serial transplanted tumor cells with 100 cells from the original mouse (P0) to cells from a next passage (P1) is shown in the curve plot (right). **b** U87MG-r#10 of different passages was extracted, and the qPCR detection of gene expression was shown in the bar graph. **c** Western blots of SOD2, MGMT, TIC-feature biomarkers were detected in naïve (GBM#4) and resistant (GBM #1) primary tumor. **d** CD133 was used as the marker for sorting. Western blot was applied for protein expression studies in subsets. **e** The tumor of different passages was extracted, and the qPCR detection for CD133 and SOD2 mRNA in the serially transplanted GBM#1 tumors was shown in the bar graph. **f** The qPCR detection of SOD2 in attached or spheroid cultures of U87MG cells. **g** Western blotting for proteins related to TIC features in sphere and the attached (Con) U87MG cells. (*P < 0.05, ***P < 0.001)

Acquired resistance was previously reported to be related to the presence of TICs that withstand treatment effect [7]. To investigate the TIC features of the resistant cells, in vivo ELDA was applied. The result showed that comparing to the parental one, U87MG-r#10 expressed higher frequency of cells possessing self-renewal ability (Additional file 5: Figure S5A and Fig. 3a left). In addition, we found the later passage (P1) of U87MG-r#10, but not the parental U87MG, thrived and showed higher growing capacity than the original one (P0) in the serial transplantation (Fig. 3a right) of the tumor. Regarding the advantage of the TIC-featured cells to enrich in the serially transplanted tissue, this would suggest the resistant tumor having higher regenerative potential and the self-renewal capacity [26]. Supportively, the mRNA expression of the later passage U87MG-r#10 showed higher expression of the TIC-associated biomarkers CD133, Bmi-1 and, SOX2 (Fig. 3b). Interestingly, the study also demonstrated that SOD2 was higher in the P1 cells, suggesting its relation to the TIC features. Next, the clinical tumor-derived primary cells were applied. We found the resistant tumor (GBM#1), which expressed significant TIC biomarkers, also had higher SOD2 expression (Fig. 3c). In those cells, the expression of SOD2 was significantly higher in CD133^+^ subsets than in CD133^−^ ones (Fig. 3d). Serial transplantation of the resistant primary cells also showed enhanced SOD2 and CD133 mRNA in the later passage (P1, Fig. 3e). Finally, in vitro spheroid assays was used to enrich the TIC subsets in U87MG-r#10, showing increased SOD2 mRNA and protein levels (Fig. 3f and g). Altogether, these studies suggested association of SOD2 and the specific subsets with the TIC features.

### SOD2 contributed to TICs that was related to TMZ resistance

**Fig. 4:**
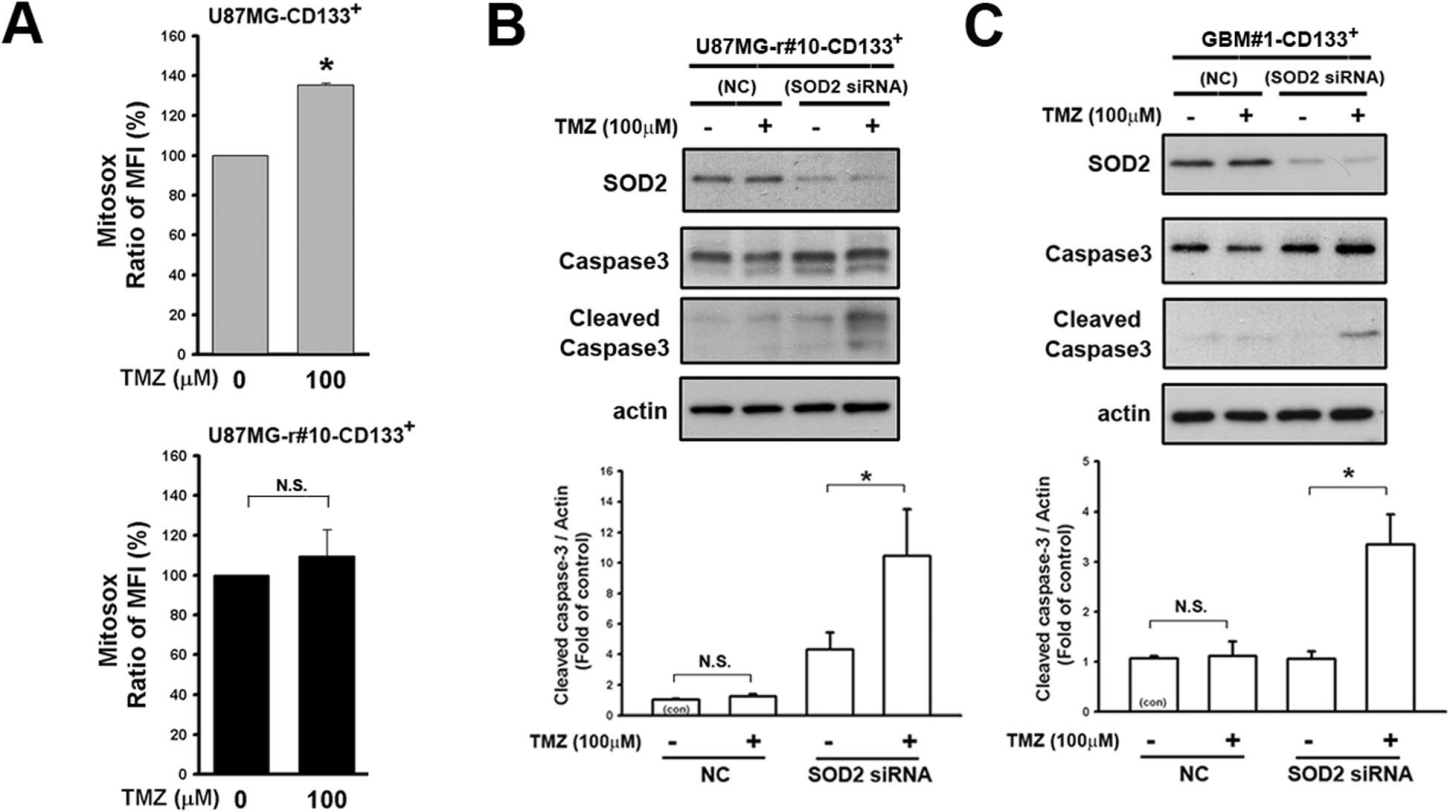
Enhancement of SOD2 function affected TMZ-related ROS generation and apoptosis. **a** The MitoSox result of CD133^+^ cells from parental and resistant U87MG cells was presented. The mean fluorescent intensity (MFI) gated by the control was calculated into the ratio over the parental cells. The average of triplicate experiment was shown in the bar graphs. **b**&**c** Level or the expression status of caspase 3 was detected by Western blotting in (**b**) CD133^+^ U87MG-r#10 and (**c**) primary tumor GBM#1. SOD2 was knocked down before treatment with TMZ. (*P < 0.05; N.S., non-significant)

We next investigated the role of SOD2 in the TICs. The mitochondrial ROS was excessively produced in CD133^+^ cells of parental U87MG 24 h after TMZ treatment, but was in less extent for those of resistant derivation (Fig. 4a & Additional file 3: Figure S3B, the treatment changes MFI to the ratio of 135.3 and 109.5% comparing to their own untreated groups mean in parental cells and in resistant cells, *P* < 0.001 and 0.52, respectively). In the CD133^+^ resistant cells, downregulation of SOD2 by siRNA resulted in increased cleavage form of caspase 3 after TMZ treatment (Fig. 4b, U87MG-r#10; Fig. 4c, GBM#1). In contrast, the cleavage protein was not or enhanced in less extent by TMZ in the control. These suggested the crucial role of SOD2 in the cells with TIC features, allowing them to survive the effects of TMZ.

**Fig. 5:**
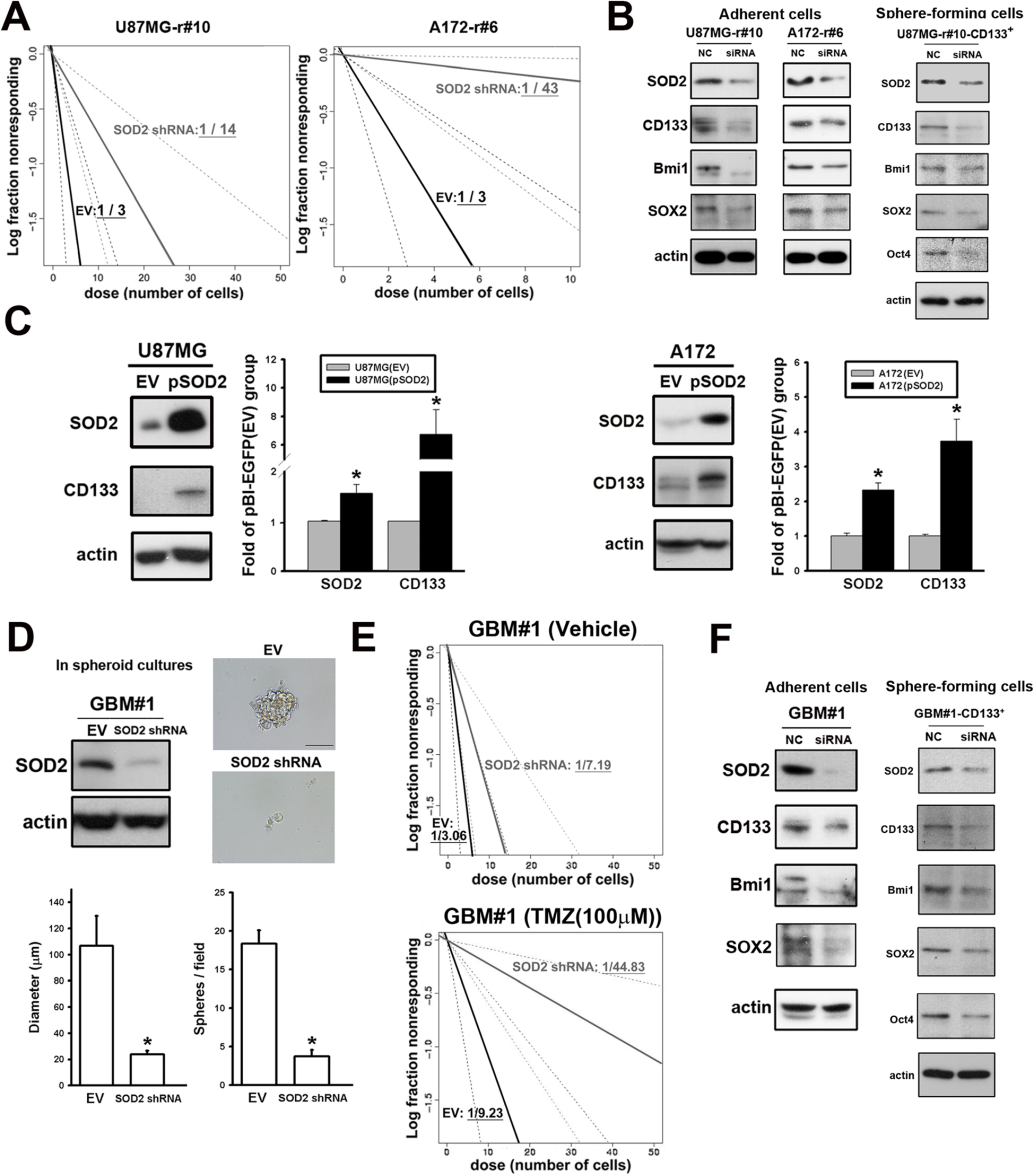
SOD2 modulation affected drug susceptibility and TIC features. **a** The frequency of the TIC-featured population in U87MG or A172 resistant cells was estimated using the in vitro extreme limiting dilution assay. **b** The cells were transfected with SOD2 siRNA and the indicated proteins were detected by western blotting. **c** Detection of CD133 level in SOD2-overexpressing U87MG or A172 parental cells was done with Western blotting. (*P < 0.05) (**d**) The SOD2 expression of the resistant primary tumor cells (GBM#1) transfected by shRNA lentiviral vector was detected with Western blotting (left upper panel). The spheroid assay was applied with the control and the knockdown cells cultured in 0.3% methylcellulose and the serum-free medium until formation of the sphere (The scale bar is 1000 μm). The diameter and the number of the spheroid cells were calculated in the bar graphs. **e** The frequency of the TIC-featured population of GBM#1 was estimated using the in vitro extreme limiting dilution assay. **f** The indicated protein levels in GBM#1 cells (left) and the CD133^+^ subsets (right) transfected with SOD2 siRNA were detected with Western blotting

We then studied the impact of SOD2 to the TIC features. Using the in vitro ELDA assay, knockdown of the SOD2 gene resulted in a lower fraction of cells with TIC features in resistant cell lines, and was more likely to form flawed spheroid colonies (Fig. 5a). In agreement, downregulation of SOD2 by siRNA decreased CD133, Bmi-1, and SOX2 expression in all the resistant cell lines examined and Oct4 in the CD133^+^ subsets, in which the TIC features were enriched (Fig. 5b). On the contrary, SOD2 overexpression in the parental cells resulted in increased CD133 expression (Fig. 5c). For validation of the TIC properties, the study was applied in primary cells. In accordance with above, SOD2 knockdown in GBM#1 resulted in worse ability for spheroid formation (Fig. 5d). The frequency of TICs fell significantly with downregulation of SOD2 in presence of TMZ treatment (Fig. 5e and Additional file 5: Figure S5B). As expected, the expression of TIC markers was decreased in siRNA-treated cells (Fig. 5f).

### Inhibition of the ROS scavenger rescued the TMZ effect in the resistant GBM

**Fig. 6:**
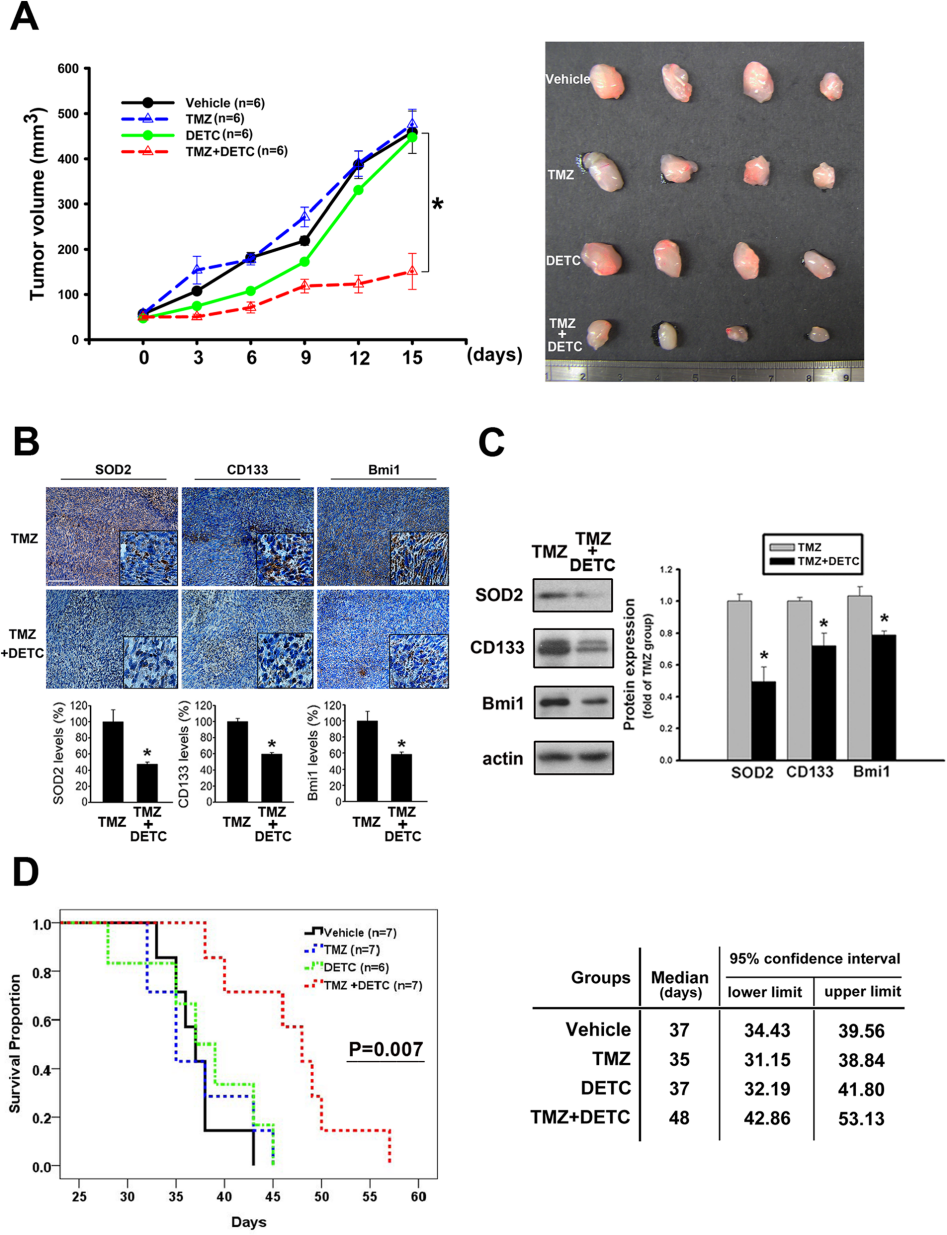
Sodium diethyldithiocarbamate trihydrate (DETC) reduced TIC features and rescued the TMZ treatment effect. **a** Mice that received subcutaneous injection of the primary tumor GBM#1 in flank area were randomly treated with TMZ or TMZ/DETC everyday. The tumor growth was plotted in the left panel. Representative images of tumor size were also shown in the right panel. **b** The representative IHC staining of the specific protein were shown in the upper panel. The levels of the detected antigen labeling were analyzed and showed in the bar graphs (scale bar: 1000 μm). **c** The lysates of the GBM#1 tumor were collected with lysis buffer. The expression of the specific proteins were studied by western blotting and calculated for the density in the bar graph. **d** Mice that received brain injection of the primary tumor GBM#1 as orthotopic model were randomly treated with TMZ or TMZ/DETC consecutively. The survival data was plotted as the Kaplan-Meier curves shown in the plot. (*P < 0.05)

The role of ROS scavengers in TMZ resistance led us to propose a strategy that involved the addition of an SOD inhibitor to enhance the sensitivity of cells to TMZ. For this purpose, DETC was applied, which induced a similar inhibitory effect on tumors in vitro as in our above RNAi studies (Additional file 6: Figure S6A). The compound was then administered intraperitoneously to the subcutaneous xenograft models and treated with TMZ. This resulted in slower tumor growth in the groups that received cotreatment of DETC and TMZ as compared with those that received TMZ alone (Fig. 6a for GBM#1 and Additional file 5: Figure S5B for U87MG-r#10). The retrieval of TMZ susceptibility was thus suggested and was accompanied by significant attenuation of SOD2, CD133, and Bmi-1 expression by IHC in the extracted tissue (Fig. 6b for GBM#1 and Additional file 6: Figure S6B for U87MG-r#10). The samples were further analyzed by western blotting, which also showed decreased expression of the aforementioned proteins (Fig. 6c). Next, a survival study with intracranial-implanted orthotopic model was applied to associate with GBM course. Treatment with combined DETC and TMZ resulted in prolonged median survival of 48 days comparing to 37 days for TMZ only (95% confidence interval: 42.9 to 53.1 versus 32.2 to 41.8, respectively). The survival curves showed significant difference in combined treatment groups (*P* = 0.007). In summary, the strategy of combining the SOD2 inhibitor with TMZ would benefit tumor treatment through an enhancement of TMZ susceptibility and a reduction in the number of TICs.

## Discussion

Multiple mechanisms were presented to explain the failure of various anti-cancer drugs [27], and understanding the resistance would help to establish potential strategies to overcome this predicament. Our study provides important information and the first evidence that acquired TMZ resistance relies upon tight regulation of ROS, leading to enrichment of TICs in GBM. Supportively, antioxidants such as glutathione and thioredoxin were often related to resistance against chemotherapy in various cancers [28]. We further identified SOD2 as the central factor in this defensive mechanism, and the most significant ROS scavengers in GBM and in the resistant cells (Fig. 1 and Additional file 1: Figure S1A) [14]. It was not surprising for SOD2 to be crucial because it was indispensible in cell functioning such as clonogenic activity [29]. In agreement, the proliferation of the cells by cell density assay was clearly impaired in parental tumor cells by downregulation of SOD2, but not as significant in resistant cells, which the basal level was much higher (Fig. 2a and Additional file 1: Figure S1B). We further uncovered that accumulation of the protein in the TMZ resistant cells was associated with enhanced TIC features that was unfavorable for the treatment. In addition, we also showed inhibition of this pathway could mitigate cellular resistance.

The process for cells to acquire drug resistance is complicated. It is often a result of specific, characterized cells capable of evading toxicity to take advantage and enrich. For example, therapeutic resistance in targeted therapies can be caused by alterations in drug targets, such as the epidermal growth factor receptor (EGFR) T790 M mutation, which leads to gefitinib resistance in nonsmall cell lung cancer [30]. In chemotherapy, it is often difficult to define an incontrovertible single factor that is responsible. Hence, our study suggested that SOD2 is one of crucial factors related to the specific subsets in the development of TMZ resistance. This was evidenced by downregulation of SOD2 to allow the drug to regain its effect (Fig. 2a and g).

Many researchers believe that small subsets of cells with TIC features confer acquired resistance because of their tendency to withstand drug-induced cytotoxicity [31]. Studies in GBM, however, have sometimes showed incompatible results. As thus, controversies remain as to whether CD133-expressing cells are more sensitive or resistant to TMZ treatment [32]. This debate is partly resulted from limitation of biomarker studies that is less associated with the biological function regarding the complexity of TIC properties [33]. In contrast, emerging functional studies have demonstrated that stress, such as hypoxia, lead to enrichment of glioblastoma stem-like cells that have a propensity to develop TMZ resistance [34]. Supportively, neuronal stem cells, which are considered their analogues, utilize hypoxia and ROS for differentiation [35]. Though less is known about the redox status in cancer TICs, recent studies showed lower levels of ROS in the specific subsets with radiation and cisplatin resistance, suggesting contribution of superior ROS regulation [28, 36]. In the stem-like cells population of tongue squamous cell carcinoma, SOD2 was suggested to mediate its migration and invasion [37]. Our preliminary data in clinically resistant GBM specimens also suggested association of expression between SOD2 and Bmi-1, a protein related to stem cell factors and drug resistance [38] (*N* = 10, r = 0.82, *P* < 0.01, Additional file 7: Figure S7). These support us for identification of higher SOD2 level in TMZ-resistant TICs that was crucial in developing resistance. So far, however, the exact mechanisms of SOD2 to enhance CD133 or the other TIC features are not clear. Kinugasa and colleagues reported that SOD2 and the ROS modulation was determinative for epithelial-mesenchymal-transition and the cell phenotype conversion related to CD44 expression, which was known as another TIC-associated marker [39]. Animals with SOD2 knockout in erythrocyte precursors would have aberrant globin genes expression related to histone modification [40]. This would suggest the potential of ROS alteration in regulating epigenetics, and subsequently, with possibility of having effect to modulate the TIC-features [41]. More studies will be needed to elucidate the mechanism.

Even though SOD2 played a role in the acquisition of TMZ resistance in GBM cells, the process was more complicated than a single gene to take full responsibility. Regardless of our significant in vitro study, the mouse model never showed full recovery of drug susceptibility allowing animals free from tumor (Figs. 2g, 6a and d). In lung cancer that possessed oncogene addiction to the EGFR mutation, eventual development of resistance might be due to multiple mechanisms such as activation of the Ras pathway or generation of ROS involved. This would result in less dependent to a single critical pathway [42]. In addition, even the TIC subsets in GBM would have already shown a degree of heterogeneity in different genetic and epigenetic functions [43]. As thus, the extent of increased SOD2 and TIC biomarkers varied between the resistant clones, suggesting other factors also had roles (Additional file 1: Figure S1). For example, DNA repair factors other than MGMT might be highly expressed in glioma stem-like cells [44]. Alteration of the other mitochondrial enzymes such as cytochrome c oxidase subunit 4 isoform 1 were also reported to be associated with TMZ resistance [45, 46], though its expression was seemingly opposite to SOD2 here (Additional file 8: Figure S8). Finally, the expression of SOD2 was not mutually exclusive to MGMT, showing the complicated scenarios when resistance was developed (Fig. 3c). Despite of the complexity, targeting a single crucial factor remains clinically valuable. A recent early-phase trial with TMZ in combination with carboxyamidotriazole, a calcium signaling inhibitor that is known to induce ROS [47], showed promising effects in glioma treatment [48]. Our strategy with a specific drug that targets the ROS scavenging protein would thus be warranted for future studies.

Some questions remain unsolved. Regarding the biological function of SOD2 in mitochondria, it was suggested that this organelle participates in the acquisition of resistance. Indeed, in acute myeloblastic leukemia, activation of mitochondria was found to be related to drug resistance [49]. It would therefore be worthwhile to define the role of mitochondria in this scenario. In addition to the molecular aspects, more questions lie in the clinical consideration. To avoid the cross-resistance caused by enrichment of TIC subsets, earlier intervention would be a better strategy [50]. However, whether the process to enrichment of the specific subsets is related to the pre-existing refractoriness, or to the inherited plasticity that contributes to later development of resistant features, is yet to be elucidated [51]. Thus, the optimal timing for the ROS inhibitory strategy to be applied remains to be determined.

## Conclusion

**Fig. 7:**
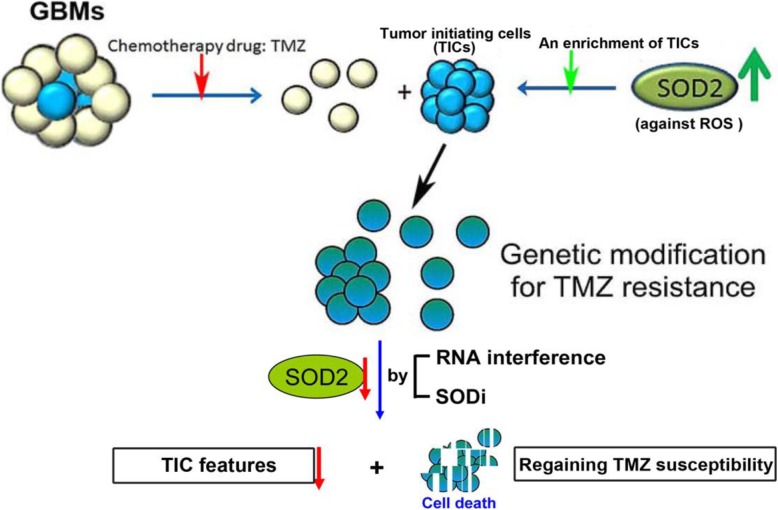
The schematic diagram illustrates the role of SOD2 in the regulation of TIC features in TMZ-resistant GBM cells. When SOD2 is down-regulated in cells with TIC features following TMZ treatment, cell apoptosis is enhanced and some TIC properties are impaired

Our study described a mechanism by which TIC subsets in GBM to protect themselves from TMZ-induced ROS and cytotoxicity by upregulation of SOD2 (Fig. 7). When SOD2 was downregulated following by TMZ treatment, cell apoptosis was enhanced with less colony formation, tumor initiation and survival. As being the crucial factor in acquiring TMZ resistance, inhibition of SOD2 can be beneficial in treatment strategy of GBM.

## Supplementary information

**Additional file 1: Figure S1.** The comparison between the parental and resistant cells. (A) The western blotting results of the parental cells and single clones of the resistant cells of U87MG (left) and A172 (right) were shown. (B) SOD2 levels were detected by Western Blot assay in the parental and resistant cells of U87MG (left) and A172 (right) with or without infection of SOD2 shRNA. The long- and the short-exposure of SOD2 plots were shown. (C) Levels of ROS scavengers in U87MG (left) or A172 (right) were compared between the parental and their resistant cells (r#10 or r#6, respectively). The detection was through qPCR for triplicated experiment with standard error shown in the bar graph.

**Additional file 2: Figure S2.** Kaplan-Meier curves of an array database from SurvExpress (http://bioinformatica.mty.itesm.mx:8080/Biomatec/SurvivaX.jsp) [18]. The original data included samples of (A) GBM from Joo KM, et al., 2013 (https://www.ncbi.nlm.nih.gov/geo/query/acc.cgi?acc=GSE42669), (B) low-grade glioma and GBM from Phillips HS, et al., 2006 (https://www.ncbi.nlm.nih.gov/geo/query/acc.cgi?acc=GSE4271), and (C) low-grade glioma and GBM from TCGA. Each line refers to cases in which SOD2 gene expression was higher or lower than the median.

**Additional file 3: Figure S3.** The mitochondrial ROS were detected with MitoSox. (A) The TMZ-induced mitochondrial ROS were in parental and resistant U87MG cells. (B) The MitoSox results of CD133+ cells from U87MG parental and resistant cells were presented. The black curves represented the unstained control, the green curves represented the untreated group, and the pink curves represented the treated group. The brackets were the mean fluorescent intensity. These plots were representative ones of the triplicate experiments.

**Additional file 4: Figure S4.** The resistant primary cells (GBM#1) were pretreated with siRNA for SOD2 knockdown. The cells were then incubated in low serum (2%) cultures medium with or without TMZ. The western blotting result of cleaved caspase 3 after TMZ treatment was shown. (*n* = 3 for each group, Data are presented as mean ± standard error, *P < 0.05).

**Additional file 5: Figure S5.** The assays of tumor-initiating cell (TIC) properties. (A) Tumor formation analysis with limiting dilution (50~1000 cells per injection) of U87-parental and TMZ-resistant (r#10) cells in subcutaneous flank area of NOD-SCID mice was performed and recorded. Tumor formation was defined as the measurement to reach 0.1 cm3 or larger. The number of tumor formation in total implanted mice was shown in the brackets. (B) The frequency of the TIC-featured population of GBM#2 (derived from another recurrent GBM patient) with or without TMZ treatment was estimated using the in vitro extreme limiting dilution assay.

**Additional file 6: Figure S6.** DETC inhibitor used *in vitro* and *in vivo*. (A) (Left) U87MG spheres were treated with different doses of DETC as indicated. The cell lysates were analyzed via western blot. The statistic results (Right) were shown. (B) Mice that received U87MG-r#10 cells were randomly treated with TMZ or TMZ/DETC for 5 consecutive days. Representative images of IHC staining in which the specific protein levels of the resistant xenografts were analyzed. *P < 0.05.

**Additional file 7: Figure S7.** The analysis from clinical samples. (A) The IHC image represented a GBM tissue section which was stained with anti-rabbit IgG as a negative control. (B) The IHC staining in the left was a representative result of SOD2 and Bmi1 from a same patient. The study was done in total of the clinical specimens from 10 patients with recurrent GBM. The area of the staining was assessed and recorded in the dotted graph at right, showing statistical correlation of SOD2 and Bmi1.

**Additional file 8: Figure S8.** The correlation between SOD2 and COX4-1. (A) Inverse correlation was noted between COX4–1 and SOD2 in GBM from a web-serve GEPIA (http://gepia.cancer-pku.cn/) using TCGA database (*P* = 0.03). (B) Levels of COX4–1 and SOD2 in U87MG (left) or A172 (right) were compared to their resistant cells (r#10 or r#6, respectively). The detection was through qPCR for triplicated experiment with standard error shown in the bar graph. (COX4–1 primers: F:5′-GAACGAGTGGAAGACGGTTG, R:5′-GGTTCACCTTCATGTCCAGC).

## Data Availability

The data generated or analyzed are included in this article, or if absent are available from the corresponding author upon reasonable request.

## Abbreviations

DETC: Sodium diethyldithiocarbamate trihydrate
EGFR: Epidermal growth factor receptor
ELDA: Extreme Limiting Dilution Analysis
FACS: Fluorescence-activated cell sorting
GBM: Glioblastoma multiforme
IHC: Immunohistochemistry
MGMT: O^6^-methylguanine-DNA methyltransferase
NGS: next-generation sequencing
PDX: Patient-derived xenograft
qRT-PCR: Quantitative real-time polymerase chain reaction
RNAi: RNA interference
ROS: Radical oxygen species
shRNA: Short hairpin RNA
SOD2: Superoxide dismutase 2
TCGA: The Cancer Genome Atlas
TIC: Tumor-initating cell
TMZ: Temozolomide
