# Supplementary material for: Enrichment of superoxide dismutase 2 in glioblastoma confers to acquisition of temozolomide resistance that is associated with tumor-initiating cell subsets
Source: J Biomed Sci. 2019 Oct 19;26:77. doi: 10.1186/s12929-019-0565-2 (PMC6800988; doi:10.1186/s12929-019-0565-2)
Supplement: Supplementary file 1 — Additional file 1: Figure S1. The comparison between the parental and resistant cells. (A) The western blotting results of the parental cells and single clones of the resistant cells of U87MG (left) and A172 (right) were shown. (B) SOD2 levels were detected by Western Blot assay in the parental and resistant cells of U87MG (left) and A172 (right) with or without infection of SOD2 shRNA. The long- and the short-exposure of SOD2 plots were shown. (C) Levels of ROS scavengers in U87MG (left) or A172 (right) were compared between the parental and their resistant cells (r#10 or r#6, respectively). The detection was through qPCR for triplicated experiment with standard error shown in the bar graph. [file 12929_2019_565_MOESM1_ESM.pdf]

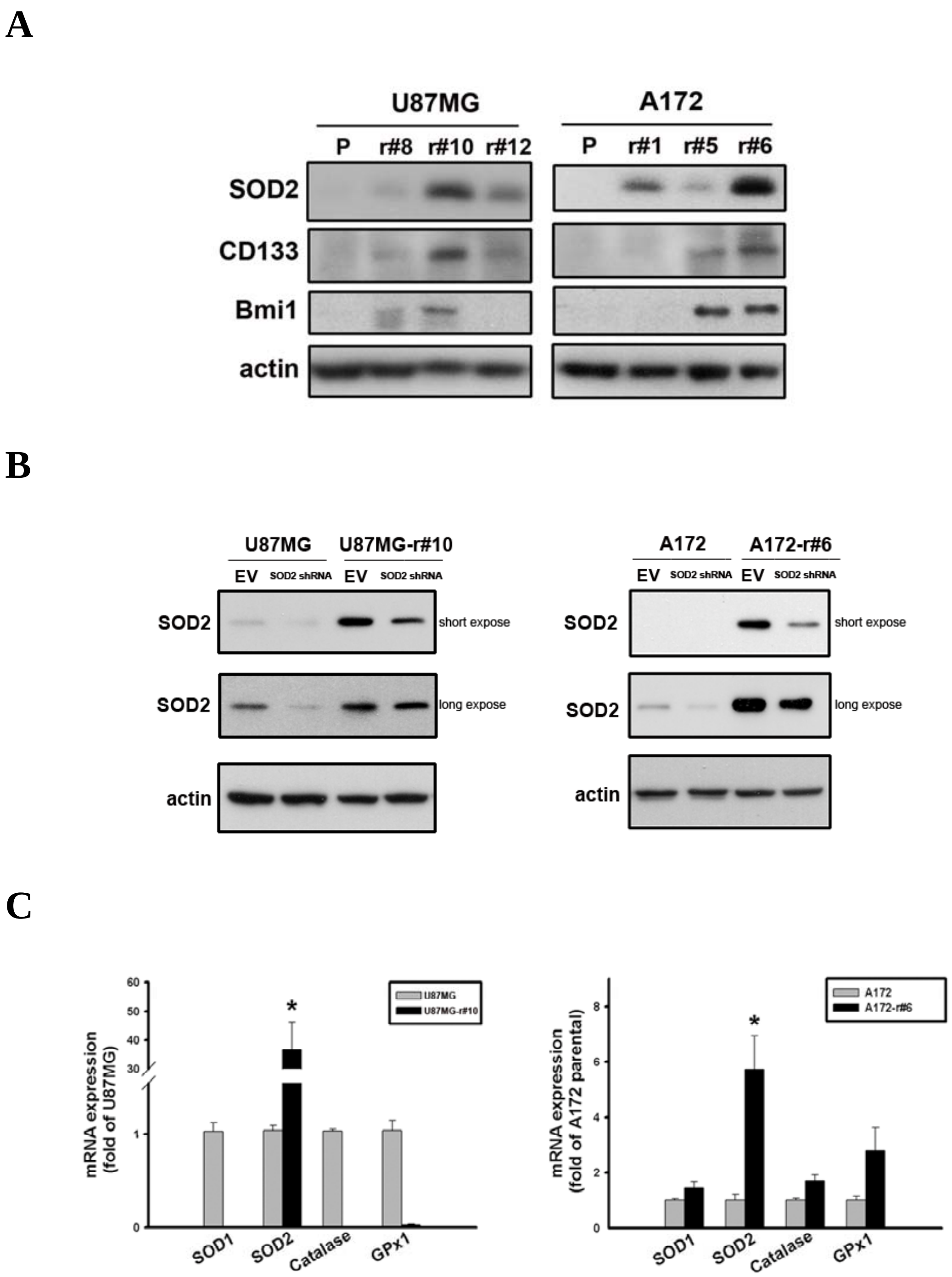

Additional file 1: Figure S1. The comparison between the parental and resistant cells. (A) The western blotting results of the parental cells and single clones of the resistant cells of U87MG (left) and A172 (right) were shown. (B) SOD2 levels were detected by Western Blot assay in the parental and resistant cells of U87MG (left) and A172 (right) with or without infection of SOD2 shRNA. The long- and the short-exposure of SOD2 plots were shown. (C) Levels of ROS scavengers in U87MG (left) or A172 (right) were compared between the parental and their resistant cells (r#10 or r#6, respectively). The detection was through qPCR for triplicated experiment with standard error shown in the bar graph.
