# Supplementary material for: Enrichment of superoxide dismutase 2 in glioblastoma confers to acquisition of temozolomide resistance that is associated with tumor-initiating cell subsets
Source: J Biomed Sci. 2019 Oct 19;26:77. doi: 10.1186/s12929-019-0565-2 (PMC6800988; doi:10.1186/s12929-019-0565-2)
Supplement: Supplementary file 2 — Additional file 2: Figure S2. Kaplan-Meier curves of an array database from SurvExpress (http://bioinformatica.mty.itesm.mx:8080/Biomatec/SurvivaX.jsp) [18]. The original data included samples of (A) GBM from Joo KM, et al., 2013 (https://www.ncbi.nlm.nih.gov/geo/query/acc.cgi?acc=GSE42669), (B) low-grade glioma and GBM from Phillips HS, et al., 2006 (https://www.ncbi.nlm.nih.gov/geo/query/acc.cgi?acc=GSE4271), and (C) low-grade glioma and GBM from TCGA. Each line refers to cases in which SOD2 gene expression was higher or lower than the median. [file 12929_2019_565_MOESM2_ESM.pdf]

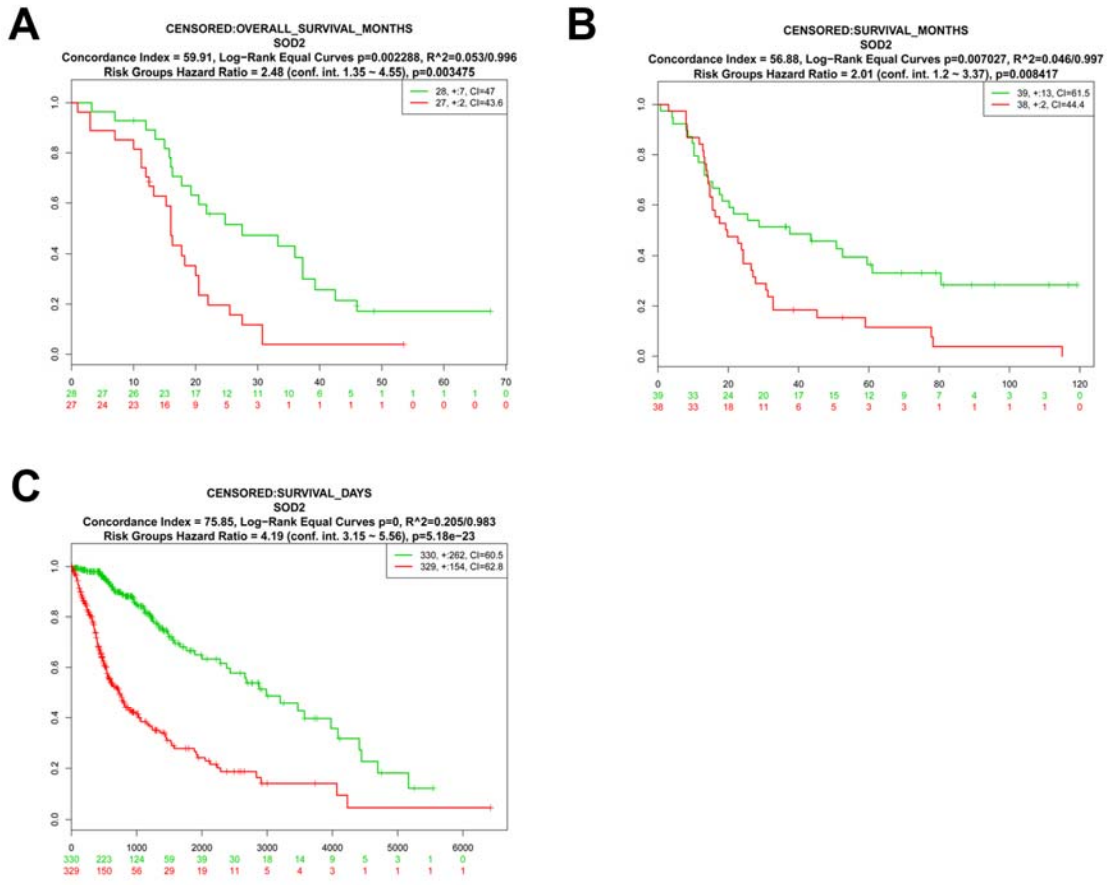

Additional file 2: Figure S2. Kaplan-Meier curves of an array database from SurvExpress (http://bioinformatica.mty.itesm.mx:8080/Biomatec/SurvivaX.jsp) [18]. The original data included samples of (A) GBM from Joo KM, et al., 2013 (https://www.ncbi.nlm.nih.gov/geo/query/acc.cgi?acc=GSE42669), (B) low-grade glioma and GBM from Phillips HS, et al., 2006 (https://www.ncbi.nlm.nih.gov/geo/query/acc.cgi?acc=GSE4271), and (C) low-grade glioma and GBM from TCGA. Each line refers to cases in which SOD2 gene expression was higher or lower than the median.
