# Supplementary material for: Enrichment of superoxide dismutase 2 in glioblastoma confers to acquisition of temozolomide resistance that is associated with tumor-initiating cell subsets
Source: J Biomed Sci. 2019 Oct 19;26:77. doi: 10.1186/s12929-019-0565-2 (PMC6800988; doi:10.1186/s12929-019-0565-2)
Supplement: Supplementary file 3 — Additional file 3: Figure S3. The mitochondrial ROS were detected with MitoSox. (A) The TMZ-induced mitochondrial ROS were in parental and resistant U87MG cells. (B) The MitoSox results of CD133+ cells from U87MG parental and resistant cells were presented. The black curves represented the unstained control, the green curves represented the untreated group, and the pink curves represented the treated group. The brackets were the mean fluorescent intensity. These plots were representative ones of the triplicate experiments. [file 12929_2019_565_MOESM3_ESM.pdf]

A

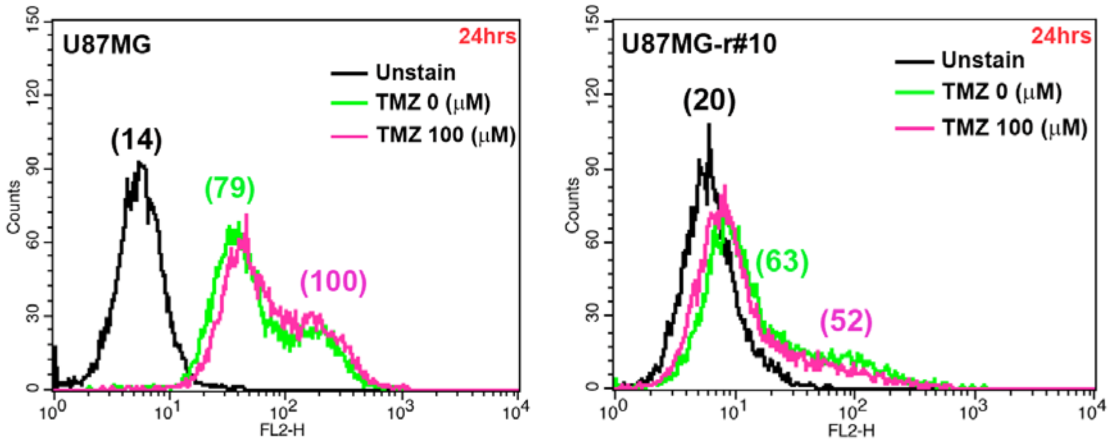

B

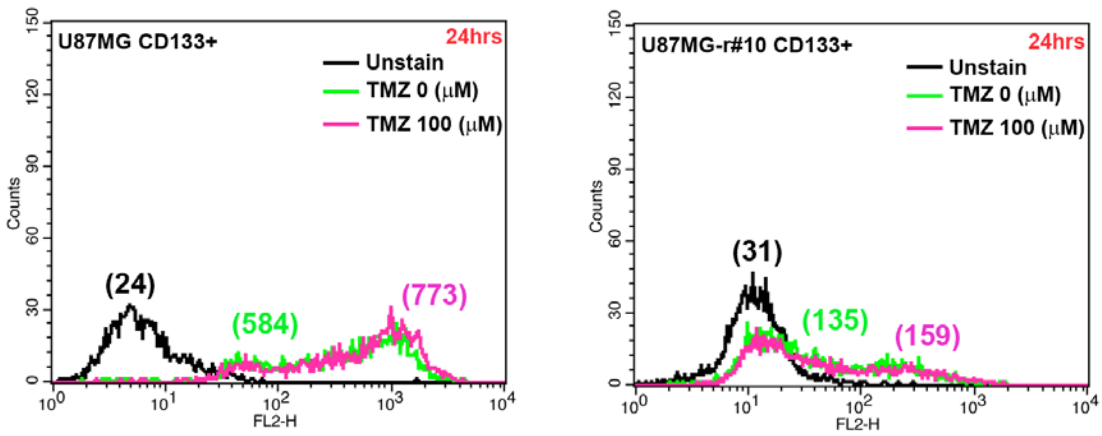

Additional file 3: Figure S3. The mitochondrial ROS were detected with MitoSox. (A) The TMZ-induced mitochondrial ROS were in parental and resistant U87MG cells. (B) The MitoSox results of $CD133^+$ cells from U87MG parental and resistant cells were presented. The black curves represented the unstained control, the green curves represented the untreated group, and the pink curves represented the treated group. The brackets were the mean fluorescent intensity. These plots were representative ones of the triplicate experiments.
