# Supplementary material for: Enrichment of superoxide dismutase 2 in glioblastoma confers to acquisition of temozolomide resistance that is associated with tumor-initiating cell subsets
Source: J Biomed Sci. 2019 Oct 19;26:77. doi: 10.1186/s12929-019-0565-2 (PMC6800988; doi:10.1186/s12929-019-0565-2)
Supplement: Supplementary file 4 — Additional file 4: Figure S4. The resistant primary cells (GBM#1) were pretreated with siRNA for SOD2 knockdown. The cells were then incubated in low serum (2%) cultures medium with or without TMZ. The western blotting result of cleaved caspase 3 after TMZ treatment was shown. (n = 3 for each group, Data are presented as mean ± standard error, *P < 0.05). [file 12929_2019_565_MOESM4_ESM.pdf]

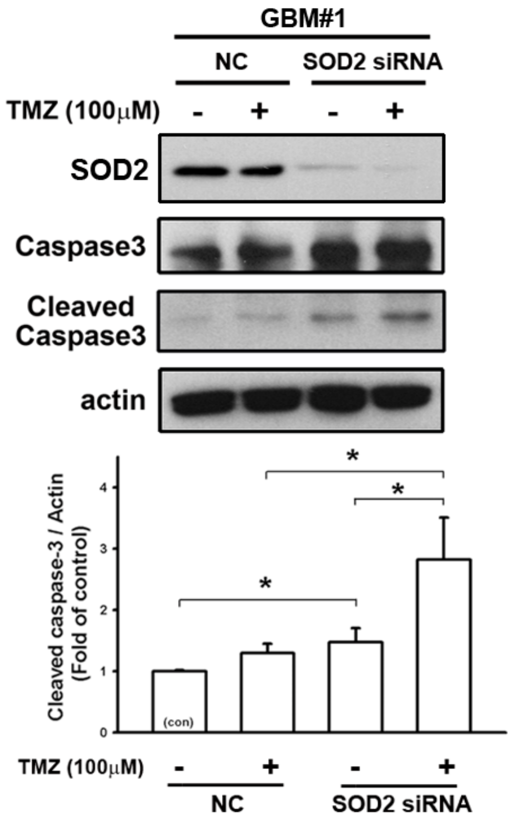

Additional file 4: Figure S4. The resistant primary cells (GBM#1) were pretreated with siRNA for SOD2 knockdown. The cells were then incubated in low serum (2%) cultures medium with or without TMZ. The western blotting result of cleaved caspase 3 after TMZ treatment was shown. (n=3 for each group, Data are presented as mean ± standard error, *P<0.05)
