# Supplementary material for: Enrichment of superoxide dismutase 2 in glioblastoma confers to acquisition of temozolomide resistance that is associated with tumor-initiating cell subsets
Source: J Biomed Sci. 2019 Oct 19;26:77. doi: 10.1186/s12929-019-0565-2 (PMC6800988; doi:10.1186/s12929-019-0565-2)
Supplement: Supplementary file 5 — Additional file 5: Figure S5. The assays of tumor-initiating cell (TIC) properties. (A) Tumor formation analysis with limiting dilution (50~1000 cells per injection) of U87-parental and TMZ-resistant (r#10) cells in subcutaneous flank area of NOD-SCID mice was performed and recorded. Tumor formation was defined as the measurement to reach 0.1 cm3 or larger. The number of tumor formation in total implanted mice was shown in the brackets. (B) The frequency of the TIC-featured population of GBM#2 (derived from another recurrent GBM patient) with or without TMZ treatment was estimated using the in vitro extreme limiting dilution assay. [file 12929_2019_565_MOESM5_ESM.pdf]

A

Cells per injection	Tumor culture	28 days	35 days	42 days	49 days	56 days	Days to growth a ~0.1 cm^3 tumor
50	U87MG	(0/5)	(0/5)	(0/5)	(0/5)	(0/5)	>56
	**U87-r#10**	(0/5)	(0/5)	**(3/5)**	(3/5)	(3/5)	56 ± 3
100	U87MG	(0/4)	(0/4)	(1/4)	(1/4)	(1/4)	>56
	**U87-r#10**	(0/4)	**(2/4)**	(4/4)	(4/4)	(4/4)	48 ± 3
250	U87MG	(0/4)	(0/4)	(0/4)	(2/4)	(3/4)	56 ± 4
	**U87-r#10**	(0/4)	(0/4)	**(3/4)**	(4/4)	(4/4)	46 ± 2
500	U87MG	(0/4)	(0/4)	(1/4)	(2/4)	(4/4)	49 ± 2
	**U87-r#10**	(0/4)	**(2/4)**	(4/4)	(4/4)	(4/4)	42 ± 2
1000	U87MG	(0/3)	(1/3)	(2/3)	(3/3)	(3/3)	46 ± 2
	**U87-r#10**	**(1/3)**	(2/3)	(3/3)	(3/3)	(3/3)	39 ± 2

B

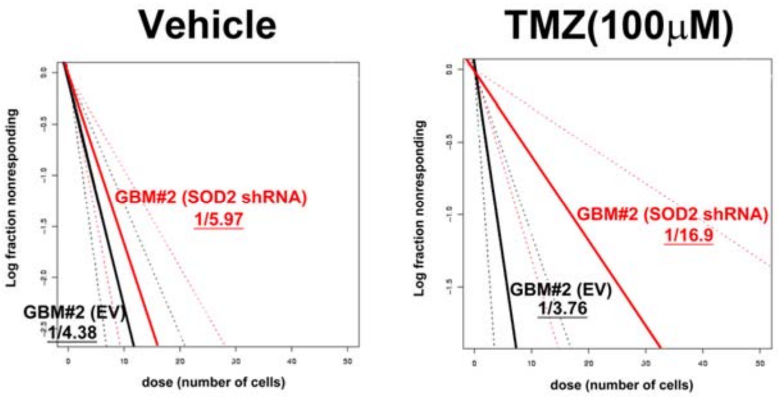

Additional file 5: Figure S5. The assays of tumor-initiating cell (TIC) properties. (A) Tumor formation analysis with limiting dilution (50~1000 cells per injection) of U87-parental and TMZ-resistant (r#10) cells in subcutaneous flank area of NOD-SCID mice was performed and recorded. Tumor formation was defined as the measurement to reach 0.1 cm^3 or larger. The number of tumor formation in total implanted mice was shown in the brackets. (B) The frequency of the TIC-featured population of GBM#2 (derived from another recurrent GBM patient) with or without TMZ treatment was estimated using the *in vitro* extreme limiting dilution assay.
