# Supplementary material for: Enrichment of superoxide dismutase 2 in glioblastoma confers to acquisition of temozolomide resistance that is associated with tumor-initiating cell subsets
Source: J Biomed Sci. 2019 Oct 19;26:77. doi: 10.1186/s12929-019-0565-2 (PMC6800988; doi:10.1186/s12929-019-0565-2)
Supplement: Supplementary file 6 — Additional file 6: Figure S6. DETC inhibitor used in vitro and in vivo. (A) (Left) U87MG spheres were treated with different doses of DETC as indicated. The cell lysates were analyzed via western blot. The statistic results (Right) were shown. (B) Mice that received U87MG-r#10 cells were randomly treated with TMZ or TMZ/DETC for 5 consecutive days. Representative images of IHC staining in which the specific protein levels of the resistant xenografts were analyzed. *P < 0.05. [file 12929_2019_565_MOESM6_ESM.pdf]

A

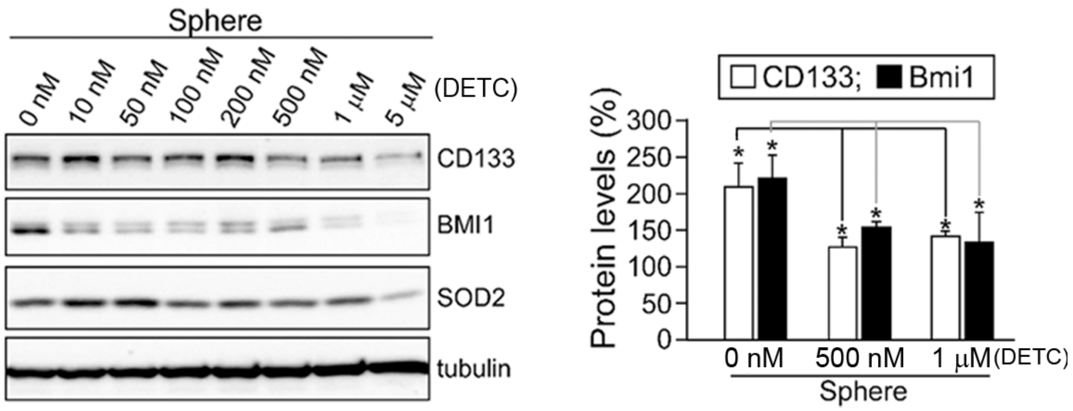

B

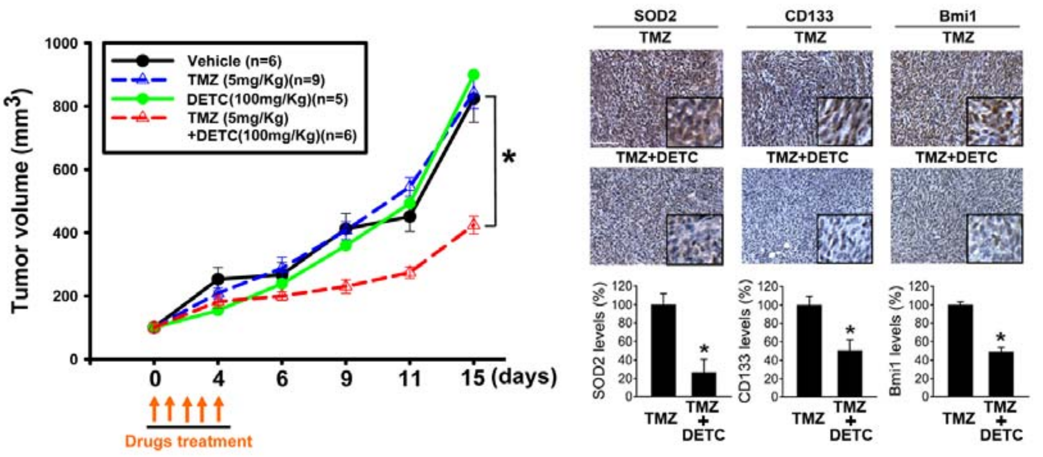

Additional file 6: Figure S6. DETC inhibitor used *in vitro* and *in vivo*. (A) (Left) U87MG spheres were treated with different doses of DETC as indicated. The cell lysates were analyzed via western blot. The statistic results (Right) were shown. (B) Mice that received U87MG-r#10 cells were randomly treated with TMZ or TMZ/DETC for 5 consecutive days. Representative images of IHC staining in which the specific protein levels of the resistant xenografts were analyzed. *P<0.05
