# Supplementary material for: Enrichment of superoxide dismutase 2 in glioblastoma confers to acquisition of temozolomide resistance that is associated with tumor-initiating cell subsets
Source: J Biomed Sci. 2019 Oct 19;26:77. doi: 10.1186/s12929-019-0565-2 (PMC6800988; doi:10.1186/s12929-019-0565-2)
Supplement: Supplementary file 7 — Additional file 7: Figure S7. The analysis from clinical samples. (A) The IHC image represented a GBM tissue section which was stained with anti-rabbit IgG as a negative control. (B) The IHC staining in the left was a representative result of SOD2 and Bmi1 from a same patient. The study was done in total of the clinical specimens from 10 patients with recurrent GBM. The area of the staining was assessed and recorded in the dotted graph at right, showing statistical correlation of SOD2 and Bmi1. [file 12929_2019_565_MOESM7_ESM.pdf]

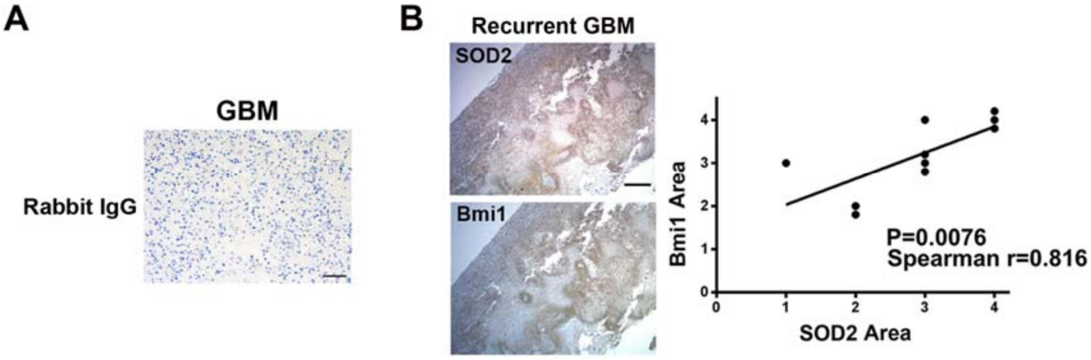

Additional file 7: Figure S7. The analysis from clinical samples. (A) The IHC image represented a GBM tissue section which was stained with anti-rabbit IgG as a negative control. (B) The IHC staining in the left was a representative result of SOD2 and Bmi1 from a same patient. The study was done in total of the clinical specimens from 10 patients with recurrent GBM. The area of the staining was assessed and recorded in the dotted graph at right, showing statistical correlation of SOD2 and Bmi1.
