# Supplementary material for: Enrichment of superoxide dismutase 2 in glioblastoma confers to acquisition of temozolomide resistance that is associated with tumor-initiating cell subsets
Source: J Biomed Sci. 2019 Oct 19;26:77. doi: 10.1186/s12929-019-0565-2 (PMC6800988; doi:10.1186/s12929-019-0565-2)
Supplement: Supplementary file 8 — Additional file 8: Figure S8. The correlation between SOD2 and COX4-1. (A) Inverse correlation was noted between COX4–1 and SOD2 in GBM from a web-serve GEPIA (http://gepia.cancer-pku.cn/) using TCGA database (P = 0.03). (B) Levels of COX4–1 and SOD2 in U87MG (left) or A172 (right) were compared to their resistant cells (r#10 or r#6, respectively). The detection was through qPCR for triplicated experiment with standard error shown in the bar graph. (COX4–1 primers: F:5′-GAACGAGTGGAAGACGGTTG, R:5′-GGTTCACCTTCATGTCCAGC). [file 12929_2019_565_MOESM8_ESM.pdf]

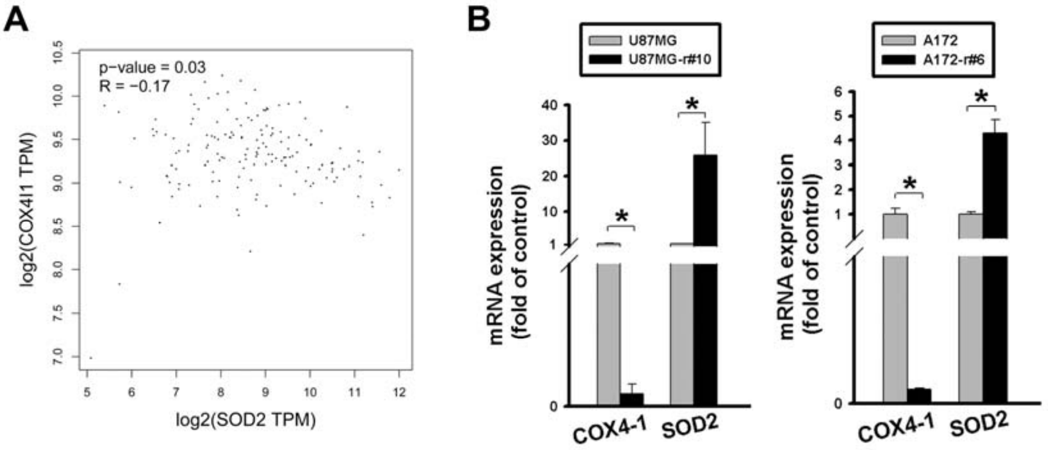

Additional file 8: Figure S8. The correlation between SOD2 and COX4-1. (A) Inverse correlation was noted between COX4-1 and SOD2 in GBM from a web-serve GEPIA (http://gepia.cancer-pku.cn/) using TCGA database (P=0.03). (B) Levels of COX4-1 and SOD2 in U87MG (left) or A172 (right) were compared to their resistant cells (r#10 or r#6, respectively). The detection was through qPCR for triplicated experiment with standard error shown in the bar graph. (COX4-1 primers: F:5'-GAACGAGTGGAAGACGGTTG, R:5'-GGTTCACCTTCATGTCCAGC).
